# Fructose-sweetened beverages induce diurnal redox dysregulation in pediatric MASLD

**DOI:** 10.1016/j.redox.2026.104012

**Published:** 2026-01-08

**Authors:** Helaina E. Huneault, Scott E. Gillespie, Zachery R. Jarrell, Shasha Bai, Ana Ramirez Tovar, Cristian Sanchez-Torres, Lucia A. Gonzalez-Ramirez, Kelsey C. Chatman, Thomas R. Ziegler, Dean P. Jones, Jean A. Welsh, Miriam B. Vos

**Affiliations:** a Department of Pediatrics, Division of Gastroenterology, Hepatology, & Nutrition, Emory University, Atlanta, GA, USA; b Department of Pediatrics and Human Development, Michigan State University, College of Human Medicine, Grand Rapids, MI, USA; c Pediatric Biostatistics Core, Department of Pediatrics, School of Medicine, Emory University, Atlanta, GA, USA; d Division of Pulmonary, Allergy, Critical Care and Sleep Medicine, Emory University, Atlanta, GA, USA; e Nutrition & Health Sciences Doctoral Program, Laney Graduate School, Emory University, Atlanta, GA, USA; f Division of Endocrinology, Metabolism and Lipids, Department of Medicine, Emory University, Atlanta, GA, USA; g Children's Healthcare of Atlanta, Atlanta, GA, USA; h Helen DeVos Children’s Hospital, Corewell Health, Grand Rapids, MI, USA

**Keywords:** Oxidative stress, Glutathione, Hepatic steatosis, Dietary sugars

## Abstract

**Background:**

Plasma glutathione/glutathione disulfide (GSH/GSSG) and cysteine/cystine (Cys/CySS) redox couples undergo diurnal variation in adults and are more oxidized in obesity-related conditions, including metabolic dysfunction-associated steatotic liver disease (MASLD). There is limited research on redox in children and no data on redox responses to sugars, despite high sugar consumption in this population. This study aimed to describe the diurnal variation of redox couples in children, assess the impact of MASLD, and evaluate responses to fructose versus glucose beverages.

**Methods:**

In a 2-day randomized, controlled, crossover feeding study, 26 children (12 with MASLD, 14 controls; aged 10–18 years) consumed isocaloric meals with fructose beverages (FB) on one day and glucose beverages (GB) (set as control) on another, following a washout period. Blood was collected every 2 h over 24 h and analyzed for Cys/CySS and GSH/GSSG. Redox potentials, E_h_(Cys/CySS) and E_h_(GSH/GSSG), were calculated using the Nernst equation. Linear mixed models assessed diurnal variation and effects of MASLD and beverage type.

**Results:**

Plasma E_h_(GSH/GSSG) and E_h_(CyS/CySS) varied significantly over time after both FB and GB (p < 0.05). With FB, E_h_(GSH/GSSG) was significantly more oxidized in children with MASLD (p = 0.034); this was not observed with GB. Among children with MASLD, FB also led to greater E_h_(GSH/GSSG) oxidation and lower GSH levels overnight (p < 0.05). While E_h_(Cys/CySS) showed a similar trend, differences did not reach statistical significance.

**Conclusions:**

Our findings demonstrate that plasma redox states vary diurnally in children and are more oxidized in those with MASLD. Fructose intake increased oxidation of the GSH/GSSG redox couple and lowered GSH concentrations overnight, indicating heightened oxidative stress. These results identify fructose as a driver of redox imbalance in pediatric MASLD and support fructose reduction and glutathione restoration as therapeutic targets.

## Highlights

• Plasma redox couples (Cys/CySS, GSH/GSSG) show diurnal variation in children.
• Children with MASLD display more oxidized plasma redox states across the day.
• Fructose intake increases oxidation of the GSH/GSSG redox couple in MASLD.
• Lower overnight GSH suggests decreased antioxidant capacity after fructose intake.
• Findings highlight fructose as a driver of redox imbalance in pediatric MASLD.

## Graphical abstract

**Figure:**
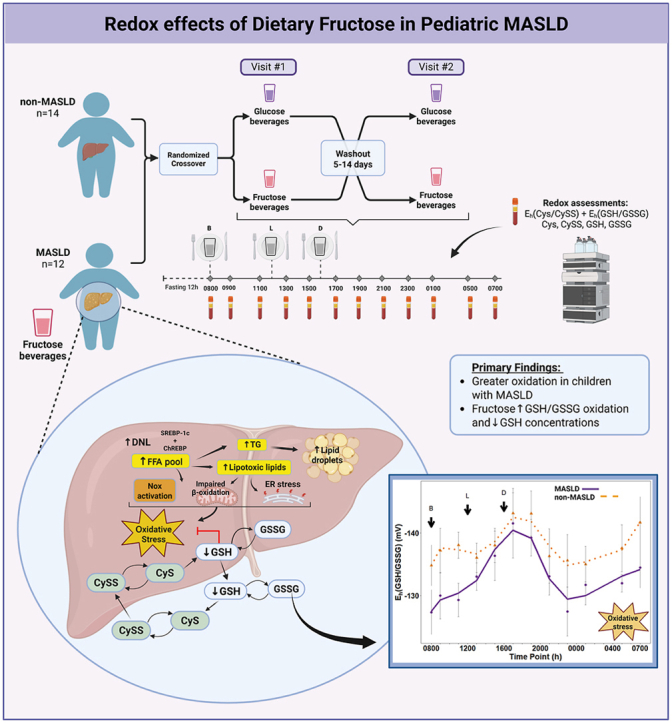

## 1 Introduction

Metabolic dysfunction-associated steatotic liver disease (MASLD), previously known as non-alcoholic fatty liver disease (NAFLD), is the most common cause of chronic liver disease in children and is a leading cause of liver-related morbidity and mortality [1,2]. MASLD is clinically characterized by hepatic steatosis (liver fat ≥5 %) along with cardiometabolic risk factors in the absence of significant alcohol consumption and other chronic liver diseases [3]. In addition, liver enzymes such as alanine aminotransferase (ALT) and aspartate aminotransferase (AST) are often elevated [4]. The disease severity exists on a spectrum that ranges from simple steatosis to metabolic dysfunction-associated steatohepatitis (MASH) and may advance to fibrosis and cirrhosis, increasing the risk of hepatocellular carcinoma [3]. While previously rare, the prevalence of MASLD has substantially increased and is now estimated at 16.5 % among all U.S. adolescents [5], including 26 % among those with obesity [6]. Notably, there are no recommended medications or supplements for treating pediatric MASLD.

The pathogenesis of MASLD is a complex, multifactorial process that is only partially understood [7]. Oxidative stress is hypothesized to play a central role in the development of MASLD and its progression to MASH [[8], [9], [10], [11], [12]]. Oxidation-reduction (redox) changes involving oxidants, including reactive oxygen species (ROS), as well as antioxidants [glutathione (GSH), vitamin E, etc.] occur in every cell in the human body as a component of ongoing metabolism [13]. When an imbalance between oxidants and antioxidants in favor of the oxidants leads to a disruption of redox signaling and control, this is known as oxidative stress [14]. To protect against oxidative stress and regulate cellular processes, such as gene transcription and protein folding, all cells have thiol-dependent antioxidant systems critical for redox regulation [[15], [16], [17]]. The reduction potentials (E_h_) for the major low-molecular weight aminothiol-disulfide redox couples, glutathione/glutathione disulfide (GSH/GSSG) and cysteine/cystine (Cys/CySS), are established indicators of systemic oxidative stress and provide a useful summary of the redox environment of human plasma [18]. Circulating E_h_(Cys/CySS) primarily reflects extracellular oxidative stress and ongoing inflammation, while E_h_^(^GSH/GSSG) is more indicative of intracellular antioxidant capacity and tissue redox status [19,20]. Recent advances in redox biology highlight that these thiol-disulfide systems are not merely biomarkers of oxidative stress but active components of redox networks that regulate metabolic homeostasis and resilience. The redox theory of aging posits that persistent oxidation of these systems signals declining physiological adaptability, a process that may begin early in life in chronic conditions such as MASLD [21]. While several studies in adult and animal models have shown that redox couples undergo diurnal variation and are more oxidized in the context of chronic disease [19,[22], [23], [24], [25]], studies measuring redox biomarker variability in children with or without MASLD are not available.

**Fig. 1:**
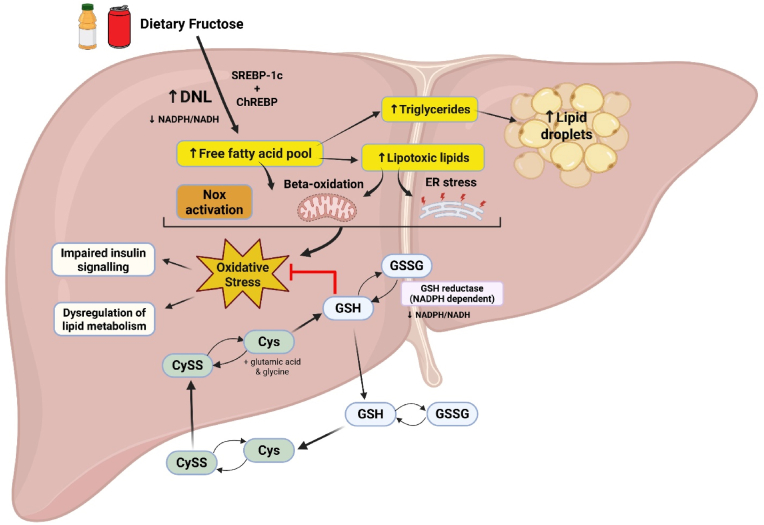
**Redox effects of dietary fructose.** In the liver, dietary fructose induces the activation of ChREBP and SREBP-1c, which transcriptionally activate lipogenic enzymes for de novo lipogenesis (DNL), an NADPH-dependent process, leading to the accumulation of free fatty acids (FFA). Excess FFAs can be converted to triglycerides, which are stored in lipid droplets or serve as substrates to produce lipotoxic lipids such as diacylglycerols and ceramides. The increased influx of FFAs can overload beta-oxidation, leading to NADPH oxidase (NOX) activation, mitochondrial dysfunction, and endoplasmic reticulum (ER) stress. Together, these processes can contribute to oxidative stress, which can modulate the activity of insulin signaling and affect the expression and activity of key enzymes in lipid metabolism, contributing to disease progression [12,28]. Major low-molecular-weight thiol-disulfide redox couples, such as glutathione (GSH)/glutathione disulfide (GSSG) and cysteine (Cys)/cystine (CySS), serve as indicators of the cellular redox state and maintain redox homeostasis by neutralizing oxidative species. GSH, an antioxidant, reduces oxidants and is converted to its oxidized disulfide form, GSSG, in the process. Importantly, recycling of GSSG back to two molecules of GSH is dependent on NADPH-dependent glutathione reductase activity, linking cellular redox balance to NADPH and NADH availability. Cys and CySS participate in the redox cycling and synthesis of GSH, along with the addition of the amino acids glutamic acid and glycine. GSH can also be exported out of hepatocytes, contributing to extracellular antioxidant defense and influencing the redox state of the surrounding environment. Thus, chronic exposure to dietary fructose may disrupt glutathione redox balance through combined effects on oxidant production and cellular reductive capacity, including reduced NADPH/NADH availability and impaired glutathione reductase activity, resulting in a more oxidized GSH/GSSG redox state. *Abbreviations:* carbohydrate response element binding protein (ChREBP); sterol regulatory element binding protein-1c (SREBP-1c), nicotinamide adenine dinucleotide phosphate (NADPH); nicotinamide adenine dinucleotide (NADH).

Dietary intake of added sugars has increased over the past few decades, and fructose, in particular, has been implicated as a contributing factor in the development and progression of MASLD through multiple mechanisms, including the promotion of oxidative stress [26]. Fructose is considered more lipogenic than glucose because its metabolism is unregulated in the liver, bypassing the rate-limiting enzyme of glycolysis and providing ample substrate supply for hepatic de novo lipogenesis (DNL) [27]. As shown in Fig. 1, dietary fructose transcriptionally stimulates hepatic DNL through the activation of sterol-regulatory element-binding protein 1c (SREBP1c) and carbohydrate-responsive element-binding protein (ChREBP) pathways [29]. When free fatty acid accumulation exceeds the liver's storage capacity, lipotoxic lipids such as diacylglycerols and ceramides are produced, promoting oxidative stress via mechanisms such as impaired mitochondrial beta-oxidation, NADPH oxidase (NOX) activation, and endoplasmic reticulum (ER) stress [12,28]. Glutathione (GSH), the major intracellular antioxidant, can reduce oxidative species by cycling with its oxidized disulfide form, GSSG [30]. In this process, GSH donates electrons to neutralize oxidants, becoming oxidized to GSSG. Cys and CySS also participate in the redox cycling and synthesis of GSH, which can be exported out of hepatocytes, contributing to extracellular antioxidant defense, and modulating the redox state of the surrounding environment [30,31]. Continual exposure to dietary fructose can result in excessive oxidant production, disrupting intracellular GSH homeostasis, leading to GSH deficiency [32].

Exploring the influence of dietary exposures such as excessive sugar intake on the variation of redox biomarkers could be a key component to understanding the complexity of MASLD pathogenesis. If fructose induces redox changes, decreasing fructose-sweetened beverage intake could be coupled with treatments replenishing GSH when treating children with MASLD. Moreover, if there is a diurnal association between the plasma redox state and MASLD development, then transient oxidation of Cys/CySS or GSH/GSSG could define a period of greatest risk.

This randomized, double-blind, two-day crossover feeding pilot study aimed to: 1) describe diurnal patterns of redox couples in children with and without MASLD, 2) determine whether MASLD influences these redox patterns, and 3) evaluate the response of redox couples to standardized meals combined with fructose-sweetened beverages (FB) compared to glucose-sweetened beverages (GB) as controls. We predicted that acute FB consumption will lead to more oxidized Cys/CySS and GSH/GSSG redox potentials than GB, with greater oxidation and altered diurnal variability among children with MASLD.

## 2 Materials and methods

***Sex as a biological variable.*** Sex was not considered as a biological variable in this study. The pediatric patient cohort in this study consisted of 19 males and seven females.

### 2.1 Study design

**Fig. 2:**
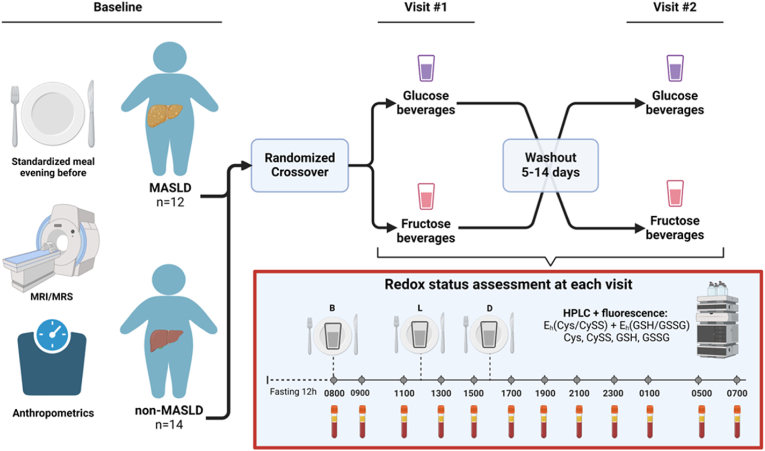
**Two-day crossover feeding study with randomization (n=26).** At baseline, all participants underwent MRI/MRS and anthropometric assessment. The evening before each visit, participants consumed a standardized meal and were randomized to receive fructose beverages (FB) or glucose beverages (GB) with three standardized meals at Visit #1 and provided with the other beverage at Visit #2. Visits #1 and #2 were scheduled at least five days apart but at most 14 days (washout). At each visit, an IV was placed for blood draws to assess redox status, and samples were drawn at 0800 h, 1 h after breakfast (B) at 0900 h, and subsequently, every 2 h until the following morning except at 0300 h to allow children to sleep. *Abbreviations:* MRI: magnetic resonance imaging; MRS: magnetic resonance spectroscopy; B: breakfast; L: lunch; D: dinner; HPLC: high-performance liquid chromatography; E_h_; redox potential; Cys; cysteine; CySS: cystine; GSH: glutathione; GSSG; glutathione disulfide.

This was a randomized, double-blind, two-day crossover feeding pilot study of acute redox changes from dietary fructose among children with and without MASLD. The two separate visit days were designed to compare responses to FB with standardized meals with isocaloric GB as the control. Participants were randomized to receive either FB or GB on the first day, followed by the alternate beverage on the second day, after a washout period ranging between 5 and 14 days. Biomarkers of oxidative stress [E_h_(Cys/CySS), Cys, CySS, E_h_(GSH/GSSG), GSH, and GSSG] were assessed every 2 h over each separate 24-h visit using high-performance liquid chromatography (HPLC) with fluorescence detection (Fig. 2). Baseline anthropometric measurements, fasting plasma lipids, glucose, insulin, and liver enzymes were also assessed at each visit. Inclusion criteria included the ability to undergo magnetic resonance imaging (MRI) or spectroscopy (MRS), and exclusion criteria were chronic illness requiring medication, including diabetes, acute illness with fever within the four weeks before the first study visit, usage of antioxidant supplements, and pregnancy.

### 2.2 Subjects

A total of 32 children aged 10–18 years were initially recruited for the study. Six participants were excluded due to missing MRI data or incomplete redox biomarker measurements, resulting in a final sample of 26 participants, including 12 with MASLD and 14 non-MASLD controls. All children attended both visits and met the eligibility criteria as described above. Subjects with MASLD (confirmed by liver biopsy within the past 2 years) were recruited from Emory Children's Center Liver Clinic, and non-MASLD subjects were recruited from the Emory Children's Center general clinics. MASLD was defined as hepatic steatosis ≥5 % by MRI or MRS performed at the first study visit, along with at least one out of five specified cardiometabolic risk factors in the absence of heavy alcohol use and other chronic liver diseases. In youth, these cardiometabolic risk factors include body mass index (BMI) ≥ 85th percentile for age/sex [BMI z-score ≥ +1], fasting serum glucose ≥100 mg/dL, blood pressure ≥130/80 mmHg, plasma triglycerides (TG) ≥ 100 mg/dL (age <10 years) or ≥ 150 mg/dL (age ≥10 years), and plasma high-density lipoprotein-cholesterol (HDL-c) ≤ 40 mg/dL(3).

### 2.3 Study visits

The evening before each of the two study visits, all participants consumed a standardized meal containing 700 kcal and 78 g sugar and then fasted for 12 h before the initial baseline measurements. Visits one and two were scheduled at least five days apart but at most 14 days apart. Anthropometric measurements, including weight and height, were performed at both visits using standard procedures. An IV was placed for blood draws at 0700, and samples were drawn at 0800 (baseline), 1 h after breakfast at 0900, and subsequently, every 2 h until the following morning, except at 0300 to allow the children to sleep. Three standardized meals (50 % carbohydrates, 30 % fat, and 20 % protein (protein 0.8 g/kg/d)) were prepared in the metabolic kitchen by a research nutritionist. Additionally, 33 % of total estimated daily calories (1.3 × estimated needs using each participant's calculated basal metabolic rate based on the Harris-Benedict formula) provided as an isocaloric, GB (dextrose control) or FB (granulated fructose dissolved in water; treatment) was divided evenly among the meals. Meals were typical for children and included items such as scrambled eggs, a hamburger, tater tots, and green beans. For each subject, lunch was served 4 h after breakfast, and dinner was served 8 h after breakfast. Typical mealtimes for a subject were 0800 (breakfast), 1200 (lunch), and 1600 (dinner), after which only *ad libitum* water was allowed (Fig. 2).

### 2.4 Materials

As described previously, materials for HPLC analysis, including sodium heparin, bathophenanthroline disulfonate sodium salt (BPDS), sodium iodoacetate, dansyl chloride, l-serine, GSH, GSSG, Cys, CySS, and sodium acetate trihydrate were purchased from Sigma Chemical Corp (St Louis, MO). Gamma-glutamyl-glutamate was obtained from MP Biomedicals Corp (Irvine, CA). Boric acid, sodium tetraborate, potassium tetraborate, perchloric acid, and acetic acid were reagent grade and were purchased locally. Methanol, acetone, and chloroform were HPLC-grade [20,22].

### 2.5 Sample collection and analysis

Complete details of the procedures used for sample collection and analysis of aminothiols in blood are reported elsewhere [22,33]. Briefly, at the bedside immediately after collection, 1.35 mL of blood was transferred to a microcentrifuge tube with 0.15 mL of a preservative solution containing 0.5 M l-serine, 9.3 mM BPDS, 0.165 M γ-glutamyl-glutamate, 0.4 M boric acid, 0.1 M sodium borate, 0.144 M sodium iodoacetate, and 2.5 mg sodium heparin/mL. After removal of blood cells, samples were incubated with sodium iodoacetate under alkaline conditions to protect free thiols and stored at −80 °C until further processing within a few weeks. These conditions were previously validated to avoid artifactual oxidation. The samples were derivatized with dansyl chloride and analyzed by HPLC with fluorescence detection [34,35]. Plasma GSH/GSSG and Cys/CySS redox states were calculated from the respective concentrations using the Nernst equation with E_o_ values of −264 mV and −250 mV for GSH/GSSG and Cys/CySS, respectively, at pH 7.4 [34]. Please see our previous publication for details on plasma lipid analysis and additional laboratory and clinical measurements [33].

### 2.6 Statistical analysis

The sociodemographic and clinical characteristics of the study participants were summarized using counts and percentages for categorical variables and means and standard deviations for continuous variables (Table 1). A small proportion of missing data (<2 %) was imputed using the R package *mice* with the predictive mean matching method [36]. The Shapiro-Wilk test was used for normality testing of the thiols and disulfides and their respective redox couples. A small number of outliers (<4 %), identified at values greater than three standard deviations from the mean, were replaced by the mean value at their respective time point. Additionally, the mean and standard error of each redox measure were calculated for each time point, and the Savitzky-Golay filter [37] was used to illustrate the signal tendency over time. All summary statistics and graphical illustrations were generated using R software version 4.2.3 (Vienna, Austria).

**Table 1:** Baseline (Visit #1) anthropometric, demographic, and clinical characteristics of the study population stratified by MASLD status.

Parameter	non-MASLD^a^	MASLD^a^	p-value^b^
**n**	14	12	
**Age (yrs.)**	13.2 (2.2)	13.2 (2.7)	0.997
**Male, n (%)**	10 (71.4 %)	9 (75.0 %)	>0.999
**Hispanic, n (%)**	8 (57.1 %)	7 (58.3 %)	0.951
**BMI**	24.7 (6.2)	33.6 (7.0)	**0.002**
**BMI Z-score**	1.1 (1.0)	2.3 (0.4)	**<0.001**
**BMI %ile**	77.4 (22.3)	98.4 (1.6)	**0.004**
**ALT (IU/L)**	20.7 (11.9)	124.2 (81.8)	**0.001**
**AST (IU/L)**	26.4 (4.9)	79.9 (55.5)	**0.007**
**TG (mg/dl)**	96.9 (51.4)	156.3 (79.6)	**0.039**
**TC (mg/dl)**	158.7 (29.5)	177.7 (31.3)	0.128
**LDL-c (mg/dl)**	98.8 (24.8)	119.9 (30.6)	0.069
**HDL-c (mg/dl)**	40.3 (13.1)	35.8 (7.0)	0.275
**NEFA (mEq/L)**	1.1 (0.3)	1.2 (0.5)	0.356
**Glucose (mg/dl)**	102.3 (14.1)	101.7 (18.2)	0.924
**Insulin (mcU/ml)**	10.0 (10.4)	42.5 (31.0)	**0.004**
**hs-CRP (mg/L)**	1.0 (1.4)	4.8 (5.4)	**0.036**
**apoB-100**	61.3 (14.9)	78.6 (21.4)	**0.029**
**Lp(a)**	18.3 (16.5)	14.6 (12.4)	0.521
**Hepatic steatosis (%)**	1.0 (1.5)	19.7 (6.5)	**<0.001**

Demographic and clinical characteristics are expressed as mean (SD) for continuous variables and count (percentage, %) for categorical variables. Welch two-sample *t* tests and Fisher's exact tests were used to compare non-MASLD and MASLD groups for continuous and categorical variables, respectively. Statistical significance was considered as p < 0.05, indicated in bold. *Abbreviations:* BMI, body mass index; ALT, alanine aminotransferase; AST; aspartate aminotransferase; TG, triglycerides; TC, total cholesterol; LDL-c, low density lipoprotein cholesterol; HDL-c, high density lipoprotein cholesterol; NEFA, nonesterifed fatty acids; hs, high-sensitivity; Lp(a), Lipoprotein(a).

a n; Mean (SD); n (%).

b Welch Two Sample *t*-test; Fisher's exact test.

To analyze the effects of time, sugar-sweetened beverage, and MASLD status on plasma redox states, repeated-measures linear mixed-effects models were implemented using SAS software version 9.4 (Cary, NC). Random intercepts were included to account for subject-level variability. Fixed effects included time, beverage type (FB vs. GB), and disease state (MASLD vs. non-MASLD), along with two two-way interactions (time × disease state, time × beverage type). Potential carryover effects were evaluated by incorporating treatment sequence (beverage order) and period (visit) as fixed effects. Model assumptions, including normality of residuals and homoscedasticity, were checked to ensure validity. Statistical significance was defined as p < 0.05. Mixed-effects models were implemented using the PROC MIXED procedures in SAS.

### 2.7 Study approvals

The study was conducted in the Emory University Hospital Clinical Research Center of the Georgia Clinical and Translational Science Alliance, Atlanta, GA, and reviewed and approved by the Emory University and Children's Healthcare of Atlanta Institutional Review Board (IRB00001550). Parents provided written informed consent, and children provided assent before the study's initiation, and research was conducted in accordance with both the Declarations of Helsinki and Istanbul.

## 3 Results

### 3.1 Subject characteristics

The clinical and demographic characteristics are summarized in Table 1. The pediatric population consisted of 26 participants, aged 10 to 18 (73.1 % males and 57.7 % identified as Hispanic). The mean BMI z-score of subjects with MASLD was 2.3 (±0.4), whereas non-MASLD controls had a mean BMI z-score of 1.1 (±1.0). Additionally, subjects with MASLD had significantly higher ALT (p = 0.001), AST (p = 0.007), triglycerides (p = 0.039), insulin (p = 0.004), high sensitivity C-reactive protein (hs-CRP) (p = 0.036), and apoB-100 levels (p = 0.029) than non-MASLD controls.

### 3.2 Diurnal variations in plasma Cys/CySS redox potential in response to fructose beverages in children with and without MASLD

**Fig. 3:**
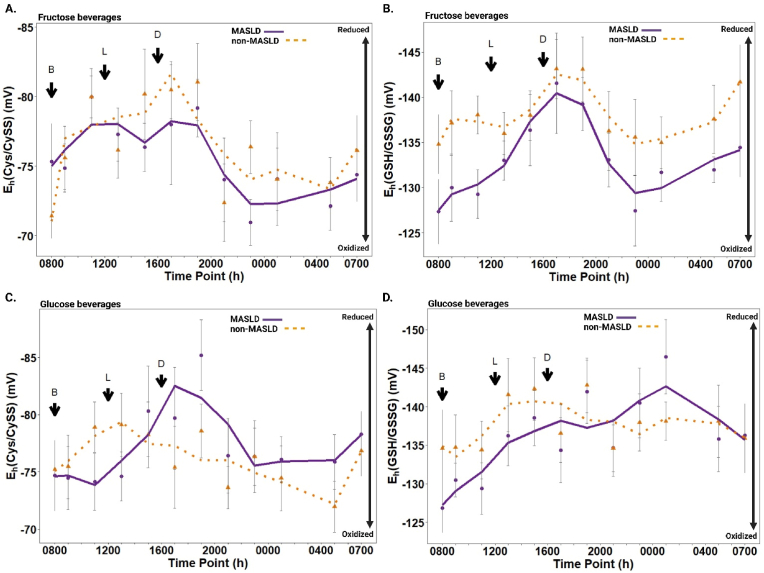
**Diurnal variation in plasma redox states among children with and without MASLD (n=26). (A)** E_h_(Cys/CySS) variation and **(B)** E_h_(GSH/GSSG) variation in response to fructose beverages; **(C)** E_h_(Cys/CySS) variation and **(D)** E_h_(GSH/GSSG) variation in response to glucose beverages. The scales for E_h_(Cys/CySS) and E_h_(GSH/GSSG) are oriented with the most reduced (most negative) values on top and the most oxidized (least negative) values on the bottom. Values are expressed as least-squares means for MASLD (n = 12) and non-MASLD (n = 14) with standard error bars at each respective time point. *Abbreviations:* B, breakfast; L, lunch; D, dinner.

In children without MASLD, the plasma Cys/CySS redox potential demonstrated significant temporal variation in response to FB (p = 0.002), with values ranging from −72 mV (mV) (most oxidized at 0800) to −82 mV (most reduced at 1900). In contrast, children with MASLD did not show significant temporal variation in E_h_(Cys/CySS) (p = 0.140), with values ranging from −71 mV (most oxidized at 2300) to −80 mV (most reduced at 1100) (Fig. 3A, Tables S1 and S2A). Both groups exhibited a general diurnal trend, becoming more reduced (more negative E_h_ values) during the daytime and more oxidized (less negative E_h_) in the evening and overnight hours.

An overall effect of time was observed across both groups (p < 0.001, Table S2A), indicating significant temporal variation in E_h_(Cys/CySS) independent of disease state. However, neither the effect of disease state (MASLD vs. non-MASLD) (p = 0.603, Table S2A) nor the interaction between disease state and time (p = 0.575) were significant, suggesting that the diurnal patterns of E_h_(Cys/CySS) did not differ significantly between groups. Although, at specific time points, children with MASLD exhibited trends toward a more oxidized E_h_(Cys/CySS) compared to non-MASLD controls. This trend began to emerge at 1500 (after lunch) and continued into the evening, becoming most apparent at 2300, where the difference between groups approached statistical significance (p = 0.102, Table S2A). These findings suggest a potential temporal shift toward a more oxidized redox state in children with MASLD during the late afternoon and evening hours.

Plasma Cys concentrations exhibited significant temporal variation in both children with MASLD (p = 0.037) and non-MASLD controls (p = 0.040). Children with MASLD generally had lower plasma Cys concentrations than non-MASLD controls overnight, particularly at time points corresponding to more oxidized E_h_(Cys/CySS); however, these differences were not statistically significant (Fig. S1A, Table S3A). Plasma CySS concentrations were consistently higher in children with MASLD at almost all time points, though these differences were also not statistically significant (p = 0.154; Fig. S1B, Table S4A). Notably, the most oxidized E_h_(Cys/CySS) values coincided with the lowest plasma Cys concentrations in both groups, highlighting the temporal relationship between Cys availability and redox potential.

### 3.3 Diurnal variations in plasma GSH/GSSG redox potential in response to fructose beverages in children with and without MASLD

In children without MASLD, plasma GSH/GSSG redox potential did not demonstrate significant temporal variation in response to FB (p = 0.538), with values ranging from −137 mV (most oxidized at 0800) to −145 mV (most reduced at 1700). Likewise, in children with MASLD, E_h_(GSH/GSSG) did not exhibit significant temporal variation (p = 0.131), with values ranging from −127 mV (most oxidized at 0800) to −142 mV (most reduced at 1700) (Fig. 3B–Tables S1 and S5A). A significant effect of period (visit) was observed in children without MASLD (p = 0.020), suggesting differences between study visits independent of time or treatment. No significant period effect was detected in children with MASLD. Similarly to E_h_(Cys/CySS), both groups exhibited a general diurnal trend, becoming more reduced during the daytime and more oxidized during the evening and overnight hours. An overall effect of time was observed across all participants (p = 0.018, Table S5A), indicating significant temporal variation in E_h_(GSH/GSSG) independent of disease state. Additionally, the main effect of disease state was significant (p = 0.034), with children with MASLD demonstrating a trend toward more oxidized E_h_(GSH/GSSG) compared to non-MASLD controls across all time points. Differences at specific time points approached significance, including 1100 (p = 0.055) and 2300 (p = 0.072, Table S5A). The interaction between disease state and time was not significant (p = 0.977), suggesting that the diurnal variation patterns of E_h_(GSH/GSSG) were generally similar between groups.

A transient period of maximal oxidation was observed in both groups during the early morning (0800) and late evening hours (2300), coinciding with the lowest plasma GSH concentrations. Plasma GSH concentrations exhibited significant temporal variation in children with MASLD (p = 0.015) but not in non-MASLD controls (p = 0.147; Fig. S2A, Table S6A). Moreover, GSH concentrations were generally lower in children with MASLD compared to non-MASLD controls at most time points, with a significant difference observed at 1100 (p = 0.021) and approaching significance at 0800 (p = 0.086) and 2100 (p = 0.093, Table S6A). In contrast, plasma GSSG concentrations did not vary significantly over time in either group (MASLD: p = 0.689; non-MASLD: p = 0.686) and were not significantly different between groups (p = 0.239; Fig. S2B, Table S7A).

### 3.4 Diurnal variations in plasma Cys/CySS redox potential in response to glucose beverages in children with and without MASLD

In response to GB, children without MASLD did not show significant temporal variation in plasma Cys/CySS redox potential, with values ranging from −73 mV (most oxidized at 0500) to −80 mV (most reduced at 1300) (p = 0.149; Fig. 3C–Tables S1 and S2B). In contrast, in children with MASLD, the time-of-day effect on E_h_(Cys/CySS) approached statistical significance with values ranging from −74 mV (most oxidized at 1100) to −85 mV (most reduced at 1900; p = 0.058).

The overall effect of time on E_h_(Cys/CySS) was significant (p = 0.010, Table S2B); however, the effect of disease state (MASLD vs. non-MASLD) with GB was not statistically significant (p = 0.869), nor was the interaction between disease state and time (p = 0.443). After dinner (1900), children with MASLD demonstrated a more reduced E_h_(Cys/CySS) compared to non-MASLD controls, though this difference did not reach statistical significance (p = 0.125). Plasma Cys concentrations mirrored the general trends in E_h_(Cys/CySS) for both groups, with no significant temporal effect observed in children with MASLD (p = 0.180, Table S3B) or non-MASLD controls (p = 0.163). There were no significant differences in Cys concentrations between groups at specific time points (all P > 0.05; Fig. S1C, Table S3B); however, the difference at 1700 approached statistical significance (p = 0.080), with higher levels in MASLD vs. non-MASLD. Plasma CySS concentrations similarly showed no significant temporal variation in either group (MASLD: p = 0.161; non-MASLD: p = 0.106), and no significant differences were detected between groups at specific time points (P > 0.05, Fig. S1D, Table S4B).

### 3.5 Diurnal variations in plasma GSH/GSSG redox potential in response to glucose beverages in children with and without MASLD

In response to GB, the plasma GSH/GSSG redox potential demonstrated significant temporal variation in children with MASLD (p < 0.001), with the most oxidized values observed in the morning (−127 mV at 0800) and the most reduced values occurring overnight (−147 mV at 0100). In contrast, children without MASLD did not exhibit significant temporal variation in E_h_(GSH/GSSG), with values ranging from −137 mV at 1100 to −145 mV at 1900 (p = 0.373; Fig. 3D–Table S5B). A significant period (visit) effect was observed in non-MASLD controls (p = 0.046), suggesting differences between visits. No significant period effect was detected in children with MASLD (p = 0.424).

Although the main effect of disease state (MASLD vs. non-MASLD) was not significant (p = 0.453), a significant overall time effect across all participants (p < 0.001) was observed, indicating diurnal fluctuations in E_h_(GSH/GSSG). The interaction between disease state and time was not significant (p = 0.670), suggesting that the overall diurnal patterns of E_h_(GSH/GSSG) were generally similar between groups with GB (Table S5B). Notably, the greatest oxidation of E_h_(GSH/GSSG) occurred at 0800 in children with MASLD and at 1100 in children without MASLD.

Plasma GSH concentrations varied significantly over time in children with MASLD in response to GB (p = 0.040) but not in non-MASLD controls (p = 0.069; Fig. S2C, Table S6B). Moreover, GSH levels were consistently lower in children with MASLD compared to controls, particularly during daytime hours, although differences at specific time points did not reach statistical significance. Plasma GSSG concentrations showed no significant temporal variation in children with MASLD (p = 0.280) or in non-MASLD controls (p = 0.145; Fig. S2D, Table S7B). Notably, a period (visit) effect was observed in non-MASLD controls (p = 0.005), suggesting variability between visits.

### 3.6 The effects of fructose beverages versus glucose beverages on diurnal variations in plasma Cys/CySS redox states among children with and without MASLD

**Fig. 4:**
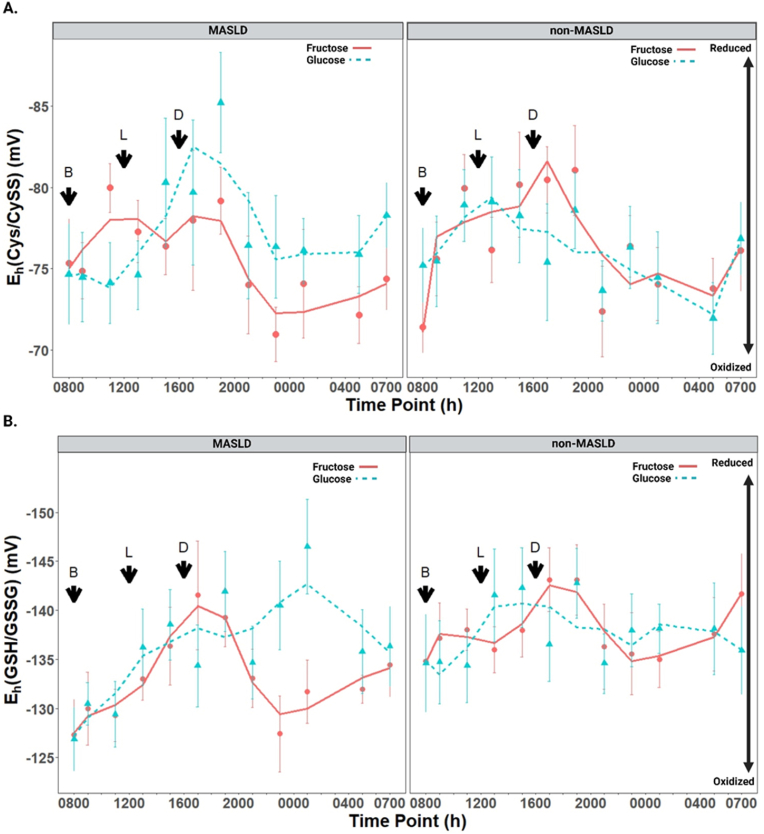
**Differences in the diurnal variation of plasma redox states between fructose and glucose beverages among children with and without MASLD (n=26). (A)** E_h_(Cys/CySS) variation in response to fructose and glucose beverages in children with MASLD (first panel) and without MASLD (second panel); **(B)** E_h_(GSH/GSSG) variation in response to fructose and glucose beverages in children with MASLD (first panel) and without MASLD (second panel). The scales for E_h_(Cys/CySS) and E_h_(GSH/GSSG) are oriented with the most reduced (most negative) values on top and the most oxidized (least negative) values on the bottom. Values are expressed as least-squares means for MASLD (n = 12) and non-MASLD (n = 14) with standard error bars at each respective time point. *Abbreviations: B, breakfast; L, lunch; D, dinner.*

In children with MASLD, the overall treatment effect of FB vs. GB on the Cys/CySS redox potential was not significant (p = 0.499), nor was the treatment-by-time interaction (p = 0.343; Fig. 4A, Table S2C). However, at specific time points, trends suggestive of increased oxidative stress with fructose treatment were observed. For example, at 1900, the Cys/CySS redox potential was more oxidized with FB compared to GB, with a difference approaching statistical significance (p = 0.120). This trend continued overnight until 0700. The beverage treatment effect on Cys concentrations was also not significant (p = 0.485: Fig. S3A, Table S3C). At certain time points, FB treatment exhibited lower Cys levels and was significantly lower at 2300 (p = 0.036). Plasma CySS concentrations were not significantly affected by beverage treatment (p = 0.797) and exhibited a similar diurnal pattern with FB and GB (Fig. S3B, Table S4C).

In children without MASLD, neither the overall treatment effect (p = 0.940) nor the treatment-by-time interaction (p = 0.618) was significant in Cys/CySS redox potential (Fig. 4A–Table S2D). Furthermore, no significant differences in Cys and CySS concentrations were observed between fructose and glucose treatments, suggesting that this group maintained a fairly consistent redox balance regardless of beverage type (Fig. S3A and S3B; Tables S3D and S4D), except for higher CySS levels with glucose at 2300 (p = 0.036).

### 3.7 The effects of fructose beverages versus glucose beverages on diurnal variations in plasma GSH/GSSG redox states among children with and without MASLD

In children with MASLD, the overall treatment effect of FB vs. GB on E_h_(GSH/GSSG) was not significant (p = 0.301), but the treatment-by-time interaction approached significance (p = 0.091). At specific time points, trends suggestive of increased oxidative stress with FB were observed (Fig. 4B–Table S5C). For instance, at 2300, the GSH/GSSG redox potential was significantly more oxidized with FB compared to GB (p = 0.012), and this difference persisted overnight at 0100 (p = 0.005). Plasma GSH concentrations exhibited a significant treatment-by-time interaction (p = 0.002), indicating temporal differences between treatments. GSH levels were significantly higher with FB at 1700 (p = 0.005) but significantly lower at 2300 (p = 0.004) and 0100 (p = 0.009; Fig. S4A, Table S6C). In contrast, plasma GSSG concentrations were not significantly affected by treatment (p = 0.810) or by the treatment-by-time interaction (p = 0.878), showing a consistent pattern across both treatments (Fig. S4B, Table S7C).

In children without MASLD, the overall treatment effect of FB vs. GB on the GSH/GSSG redox potential was not significant (p = 0.999), and no treatment-by-time interaction was observed (p = 0.560; Fig. 4B–Table S5D). A significant effect of beverage sequence (beverage order) was observed (p = 0.003). Plasma GSH levels showed a significant treatment-by-time interaction (p = 0.001), but differences were only significant at 2300, where FB treatment resulted in lower GSH levels than GB (p < 0.001: Fig. S4A, Table S6D). Additionally, plasma GSSG levels demonstrated a significant beverage sequence effect (p = 0.035) but no overall treatment effect (p = 0.259) or treatment-by-time interaction (p = 0.434). FB treatment was also associated with higher GSSG levels than GB at 1100 (p = 0.027), reflecting potential early oxidative stress responses to fructose (Fig. S4B, Table S7D).

## 4 Discussion

Oxidative stress is a critical factor influencing the development and progression of MASLD; however, little is known about the diurnal variation of biomarkers of oxidative stress in children with and without MASLD. In this study, we investigated the response of plasma Cys/CySS and GSH/GSSG redox couples to standardized meals combined with FB or GB in children with MASLD compared to non-MASLD controls. Our findings revealed significant diurnal variation in redox biomarkers, with distinct patterns emerging between MASLD and non-MASLD groups. Notably, fructose consumption appeared to exacerbate oxidative stress in children with MASLD, as evidenced by significant shifts in E_h_(GSH/GSSG) and decreased GSH concentrations following FB intake. These results underscore the potential impact of dietary factors, particularly fructose, on oxidative stress regulation in pediatric MASLD and highlight the importance of considering diurnal variation when assessing oxidative stress biomarkers in this population.

### 4.1 Differences in the diurnal variation of redox biomarkers in MASLD vs. non-MASLD

The onset and progression of MASLD are closely linked to oxidative stress, driven by multiple factors such as lipotoxicity, alterations in the gut microbiome, mitochondrial dysfunction, and insulin resistance [38]. The liver plays a central role in the production and systemic distribution of low molecular weight thiols, including GSH and Cys, which are critical for maintaining redox homeostasis. Consequently, the liver is particularly susceptible to imbalances in redox status, which can lead to impaired antioxidant defense, oxidative stress, and hepatic inflammation [39].

In this study, both children with and without MASLD demonstrated considerable variation in plasma Cys/CySS and GSH/GSSG redox states; however, children with MASLD exhibited a trend towards greater oxidation, particularly during the late afternoon and evening hours, compared to non-MASLD controls. Although these differences were not statistically significant, they align with studies in adults showing disrupted circadian regulation of redox homeostasis in metabolic diseases such as type 2 diabetes and obesity [25,40]. Notably, the lack of statistical significance may be attributed to our small sample size, which limits the power to detect significant differences.

The altered diurnal variation in children with MASLD, characterized by a shift toward greater oxidation, may reflect underlying oxidative stress and disruptions in redox regulation. More specifically, the transient periods of maximal oxidation observed in the early morning (∼0800), and late evening (∼2300) suggest impaired metabolic flexibility in adapting to physiological transitions, such as postprandial oxidative stress from dietary intake and increased metabolic demands following overnight fasting. These findings emphasize the importance of timing in clinical assessments of redox biomarkers to ensure accurate evaluations and optimize intervention strategies.

Children with MASLD also had significantly lower plasma GSH concentrations than non-MASLD controls, further highlighting compromised antioxidant defenses in this group. The most oxidized GSH/GSSG redox states coincided with the lowest plasma GSH concentrations. This relationship between GSH availability and E_h_(GSH/GSSG) underscores the role of GSH depletion in driving redox imbalance in pediatric MASLD. These findings are consistent with prior studies in human and animal models that have shown decreased GSH levels in subjects with MASLD, along with increased levels of biomarkers of oxidative stress, such as malondialdehyde (MDA), lipid peroxides, 8-isoprostane, and 4-hydroxy-2-nonenal (4-HNE) [41]. Moreover, studies in adults with MASLD and MASH have shown that the GSH/GSSG redox ratio decreases with disease severity, reflecting impaired tissue antioxidant capacity [42,43]. It is important to note that oxidation of the GSH/GSSG and Cys/CySS redox couples does not exclusively reflect increased ROS formation. These couples integrate both oxidant production and the capacity to regenerate reduced thiols, which depends on NADPH-dependent reductase systems, mitochondrial function, and precursor availability [17,18]. In MASLD, the more oxidized redox potentials we observed likely reflect a combination of elevated oxidative burden and decreased reductive capacity, including impaired NADPH-dependent recycling of thiols and lower availability of GSH [43].

A recent study further supports the role of targeting oxidative stress in MASLD management, showing that a 16-week N-acetylcysteine (NAC) treatment, a derivative of cysteine and a powerful antioxidant known to increase GSH levels, was well tolerated in children with obesity and MASLD, leading to improvements in oxidative stress, inflammation, insulin resistance, and liver outcomes [44]. Other studies involving antioxidant-based treatment for pediatric MASLD, such as vitamin E and cysteamine bitartrate, have shown mixed results for improved liver function and histology [45,46]. However, a secondary analysis of the CyNCh (Cysteamine Bitartrate Delayed-Release for the Treatment of Nonalcoholic Fatty Liver Disease in Children) trial found that younger and lighter-weight children (≤65 kg) treated with cysteamine bitartrate were significantly more likely to experience improvements in liver histology and ALT levels compared to placebo [45]. These findings highlight the therapeutic potential of interventions aimed at restoring redox homeostasis, such as NAC, in pediatric MASLD, though additional work is needed to determine effective dosing and timing of administration, particularly given the heterogeneity of MASLD and the diurnal variation in redox state observed in our study [47].

### 4.2 Impact of fructose beverages on plasma redox states among children with and without MASLD

Excessive dietary fructose intake has been implicated as a driver of oxidative stress and the progression of metabolic dysfunction in individuals with MASLD (Fig. 1). Fructose is metabolized differently from glucose in the liver, bypassing phosphofructokinase and promoting unregulated substrate flux that accelerates DNL, depletes ATP, and increases mitochondrial oxidant production [48,49]. Additionally, fructose alters the gut microbiome, possibly contributing to oxidative stress and inflammation [32,50].

In our study, children with MASLD exhibited significantly lower plasma Cys and decreased GSH concentrations in response to FB consumption. This led to a trend toward more oxidized Cys/CySS and GSH/GSSG redox states compared to GB, particularly during the early morning and late evening/overnight hours. Importantly, this trend was not observed in children without MASLD, highlighting the reduced metabolic flexibility of children with MASLD to restore redox homeostasis after FB intake. These findings align with previous studies demonstrating decreased GSH concentrations in individuals with MASLD and further highlight the susceptibility of redox systems to fructose-induced oxidative stress [39,43]. Although additional oxidative stress markers were not measured in this study, prior work provides mechanistic context for the more oxidized GSH/GSSG redox state observed following fructose exposure in children with MASLD. Fructose metabolism increases cellular demand for NADPH through multiple pathways, including stimulation of DNL, an NADPH-dependent process [21,48]. Increased competition for NADPH may limit availability for glutathione reductase-mediated recycling of GSSG back to GSH, thereby constraining maintenance of reduced glutathione pools without proportional accumulation of GSSG [51]. This mechanism is consistent with fructose-feeding models reporting depletion of hepatic GSH with modest changes in GSSG and aligns with redox network frameworks in which oxidation of the GSH/GSSG redox couple reflects reduced glutathione recycling capacity rather than oxidant burden alone [10,52,53].

In addition, the E_h_(Cys/CySS) levels observed in children with MASLD following FB intake mirrored those previously reported in non-diseased adults over 60 years of age [22], suggesting that MASLD may be associated with an early emergence of redox profiles typically seen with aging [21]. Given that Cys/CySS is a biomarker of extracellular oxidative stress and ongoing inflammation [54], this degree of oxidation likely reflects an elevated inflammatory burden that could contribute to disease progression. From a clinical perspective, these results highlight the importance of decreasing high-fructose-sweetened beverage consumption in children with MASLD, particularly during vulnerable periods like breakfast, when juice is frequently consumed, and late-night snacking, when the risk of oxidative stress may be heightened. Of note, our findings should not be interpreted as supporting non-nutritive sweeteners as alternatives to sugar-sweetened beverages. Although they reduce added sugar intake, their metabolic and redox effects remain uncertain, and both the American Heart Association and the American Academy of Pediatrics advise against routine use in children due to limited long-term safety data [55].

## 5 Strengths and limitations

To our knowledge, this study was the first to measure the diurnal variation of redox couples in children with and without MASLD. Key strengths of this study include the highly controlled methodology, which included standardized meals provided the evening before testing and during sample collection, precise measurement of redox couples, and the use of same-subject controls. Redox couples were measured using a validated high-performance liquid chromatography (HPLC) method developed at Emory University, combined with immediate bedside processing. Blood samples were treated with a thiol-preserving solution and centrifuged within 2 min of collection to prevent artifactual oxidation and accurately capture plasma redox potentials. This protocol was made possible by the support of the CTSA-funded Clinical Research Unit and the expertise of experienced research nursing staff.

Our study's small sample size may have limited the power to identify differences between the MASLD and non-MASLD control groups. Although a 14-day washout was included to minimize carryover effects, the significant influence of period (visit) and beverage sequence observed with GSH/GSSG redox may reflect external factors like dietary variability, lifestyle changes, or physiological fluctuations. While these effects were accounted for through covariate adjustment in the regression models, residual confounding cannot be completely ruled out. Despite these limitations, the crossover design and high-frequency sampling provided valuable insights into diurnal redox changes, emphasizing the need for larger studies to confirm these findings. Furthermore, we analyzed the response to a high dose of fructose and glucose (33 % of total energy needs), which is not likely to be consumed on a daily basis by most children. We selected a dose of 33 % of total calories, reflecting the proportion of added sugars typically consumed by children with the highest dietary sugar intake [56,57]. Our study also lacked a control arm where water was provided with meals instead of sugar-sweetened beverages. However, we aimed to investigate the response to added sugars (fructose vs. glucose beverages), given their role in MASLD onset and progression, and because most U.S. children exceed dietary guidelines for added sugar intake, primarily through sugar-sweetened beverages [27,56]. Including a water-control condition would have required an additional 24-h inpatient visit, increased participant burden, and prevented blinded, isocaloric comparisons within the crossover design. Future studies will be needed to test whether lower fructose doses, or comparisons with water, yield different redox responses in children with MASLD.

Moreover, there was a significant difference in obesity between our MASLD and non-MASLD control groups. Obesity is also associated with oxidative stress, although during recruitment, we did not exclude overweight or obese subjects from our control group, which contained both children with obesity and normal weight status. In addition, our patient population primarily consisted of Hispanic youth, and therefore, our findings are not generalizable to MASLD patients from other ethnic backgrounds. Larger and more diverse cohorts will be needed to confirm the generalizability of these findings. Another limitation is that we did not measure additional redox biomarkers such as NAD^+^/NADH, NADP^+^/NADPH, the thioredoxin redox systems (Trx1 and Trx2), total thiols, or total antioxidant capacity, nor did we quantify markers of oxidative damage, such as 8-oxo-dG, MDA, or 4-HNE. These complementary measures would further distinguish whether the more oxidized GSH/GSSG redox couple observed in MASLD reflects increased oxidative burden, decreased reductive capacity, or both. We also did not assess NOX2 activation markers, such as sNOX2-dp, which have been reported to be elevated in MASLD [58]. Future studies incorporating a broader panel of redox and oxidative injury biomarkers will be important for clarifying the mechanisms underlying redox imbalance in pediatric MASLD.

## 6 Conclusions

This study provides novel insights into the diurnal variations of plasma redox couples in children with and without MASLD. In children with MASLD, fructose consumption was associated with greater oxidation and decreased antioxidant capacity. These findings align with studies in adults, emphasizing the role of oxidative stress in MASLD pathogenesis and the potential impact of dietary fructose. The observed diurnal variation in redox biomarkers highlights the importance of considering timing in both research and clinical care. Avoiding fructose consumption, particularly during periods of maximal oxidation, such as early morning and late evening, may help mitigate oxidative stress in children with MASLD. Future research with larger, more diverse cohorts is needed to validate these findings and guide strategies for mitigating oxidative stress in pediatric MASLD. In addition, studies are needed to evaluate GSH repletion as part of a comprehensive treatment plan for children with MASLD.

## Declaration of Generative AI and AI-assisted technologies in the writing process

During the preparation of this work, the authors used OpenAI's ChatGPT (version 4) to refine R code and assist with minor language improvements, including grammar and readability. After using this tool, the authors reviewed and edited the content as needed and take full responsibility for the content of the published article.

## Funding

This work was supported by the National Center for Advancing Translational Sciences of the National Institutes of Health under Award Number UL1TR002378 and NIH grants to MBV, including K23 DK080953, R01 DK125701, R01 NR019083, and K24 HL 171937, as well as the Georgia Clinical Translational Science Alliance (UL1 TR002378). The content is solely the responsibility of the authors and does not necessarily represent the official views of the National Institutes of Health.

## CRediT authorship contribution statement

**Helaina E. Huneault:** Formal analysis, Investigation, Methodology, Visualization, Writing – original draft, Writing – review & editing. **Scott E. Gillespie:** Conceptualization, Formal analysis, Methodology, Supervision, Visualization, Writing – original draft, Writing – review & editing. **Zachery R. Jarrell:** Formal analysis, Methodology, Supervision, Visualization, Writing – review & editing. **Shasha Bai:** Formal analysis, Methodology, Writing – review & editing. **Ana Ramirez Tovar:** Writing – review & editing. **Cristian Sanchez-Torres:** Writing – review & editing. **Lucia A. Gonzalez-Ramirez:** Writing – review & editing. **Kelsey C. Chatman:** Writing – review & editing. **Thomas R. Ziegler:** Data curation, Investigation, Project administration, Resources, Supervision, Writing – review & editing. **Dean P. Jones:** Conceptualization, Data curation, Formal analysis, Investigation, Methodology, Project administration, Resources, Supervision, Visualization, Writing – original draft, Writing – review & editing. **Jean A. Welsh:** Supervision, Writing – review & editing. **Miriam B. Vos:** Conceptualization, Data curation, Formal analysis, Funding acquisition, Investigation, Methodology, Project administration, Resources, Supervision, Validation, Visualization, Writing – original draft, Writing – review & editing.

## Declaration of competing interest

The authors declare the following financial interests/personal relationships which may be considered as potential competing interests: MBV serves as a consultant to Boehringer Ingelheim, Novo Nordisk, Eli Lilly, Intercept, Takeda, and Alberio. She has stock or stock options in Thiogenesis and Tern Pharmaceuticals. Her institution has received research grants (or in-kind research services) from Target Real World Evidence, Quest, Labcorp, and Sonic Incytes Medical Corp. The remaining authors have nothing to declare.

## Data Availability

Raw datasets generated or analyzed during the study are not publicly available but can be obtained from the corresponding author upon reasonable request.
