# Supplementary material for: Fructose-sweetened beverages induce diurnal redox dysregulation in pediatric MASLD
Source: Redox Biol. 2026 Jan 8;90:104012. doi: 10.1016/j.redox.2026.104012 (PMC12830102; doi:10.1016/j.redox.2026.104012)

Figure S1. Diurnal variation in plasma cysteine (Cys) and cysteine (CySS) concentrations among children with and without MASLD (n=26). (A) Cys variation and **(B)** CySS variation in response to fructose beverages; **(C)** Cys variation and **(D)** CySS variation in response to glucose beverages. Values are expressed as least-squares means for MASLD (n=12) and non-MASLD (n=14) with standard error bars at each respective time point. *Abbreviations:* B, breakfast; L, lunch; D, dinner.

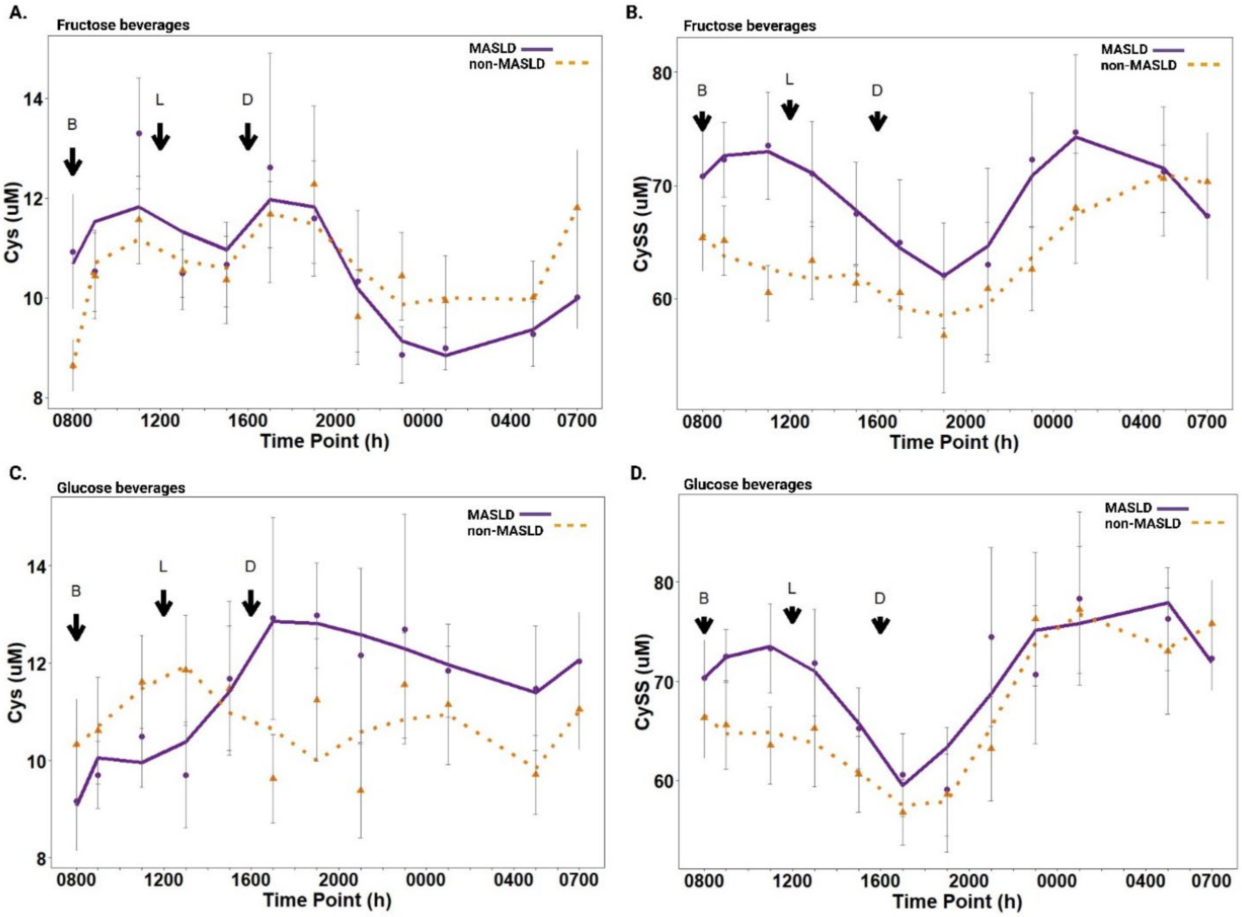

Figure S2. Diurnal variation in plasma glutathione (GSH) and glutathione disulfide (GSSG) concentrations among children with and without MASLD (n=26). (A) GSH variation and **(B)** GSSG variation in response to fructose beverages; **(C)** GSH variation and **(D)** GSSG variation in response to glucose beverages. Values are expressed as least-squares means for MASLD (n=12) and non-MASLD (n=14) with standard error bars at each respective time point. *Abbreviations:* B, breakfast; L, lunch; D, [illegible]

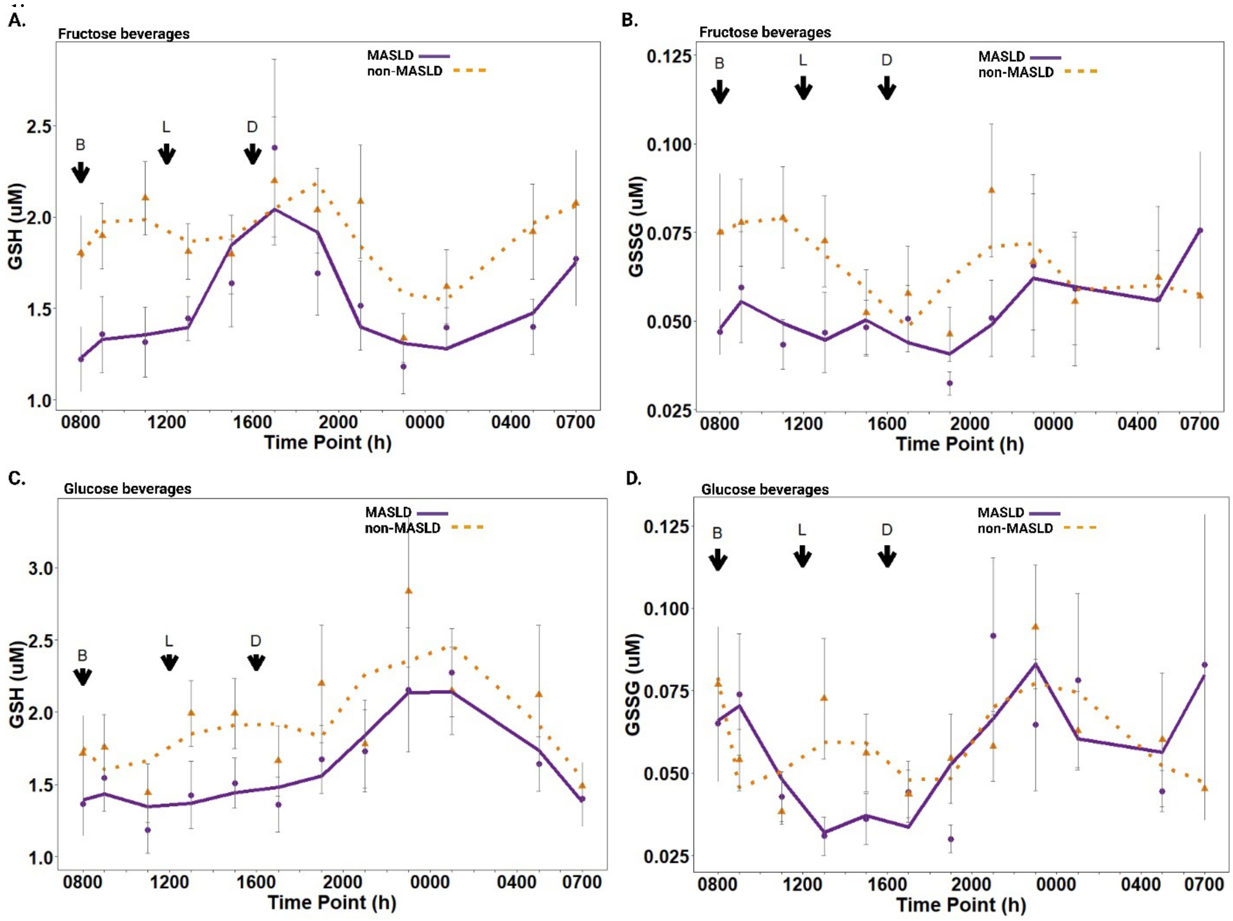

Figure S3. Differences in the diurnal variation of plasma cysteine (Cys) and cysteine (CySS) concentrations between fructose and glucose beverages among children with and without MASLD (n=26). (A) Cys variation in response to fructose and glucose beverages in children with MASLD (first panel) and without MASLD (second panel); **(B)** CySS variation in response to fructose and glucose beverages in children with MASLD (first panel) and without MASLD (second panel). Values are expressed as least-squares means for MASLD (n=12) and non-MASLD (n=14) with standard error bars at each respective time point. *Abbreviations: B, breakfast; L, lunch; D, dinner.*

A.

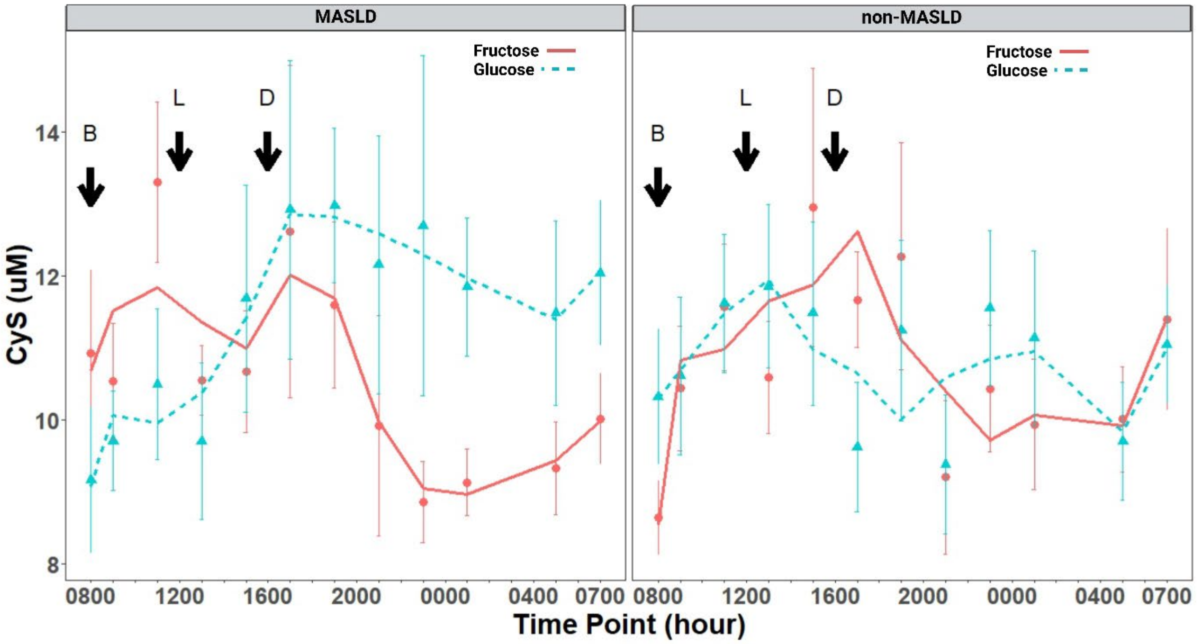

B.

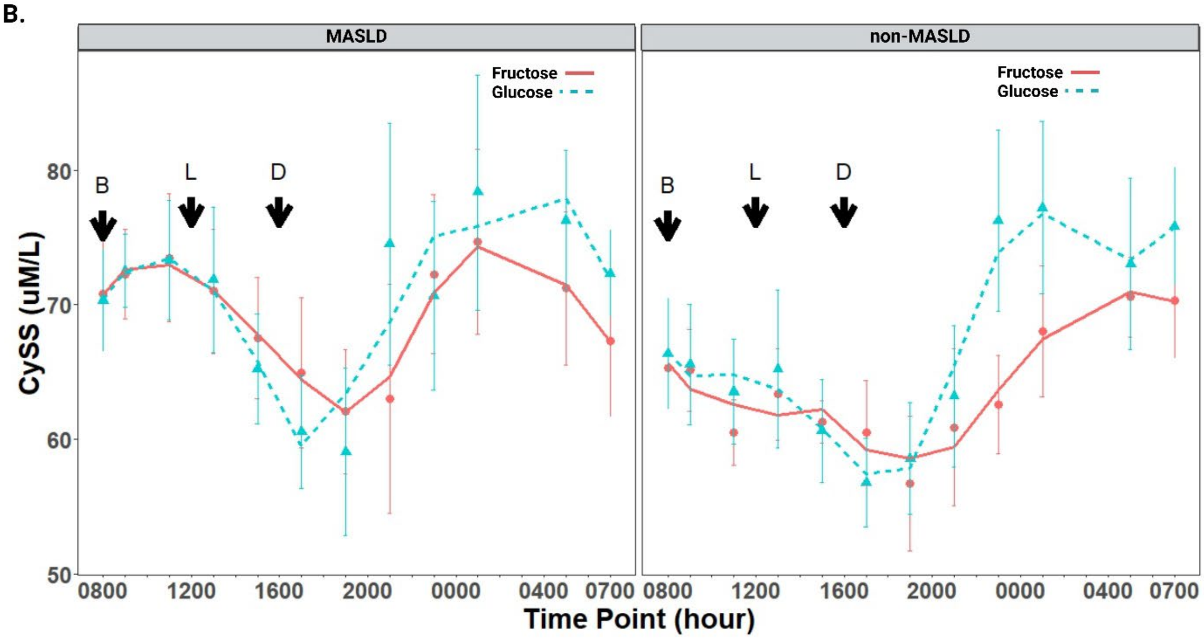

Figure S4. Differences in the diurnal variation of plasma glutathione (GSH) and glutathione disulfide (GSSG) concentrations between fructose and glucose beverages among children with and without MASLD (n=26). (A) GSH variation in response to fructose and glucose beverages in children with MASLD (first panel) and without MASLD (second panel); **(B)** GSSG variation in response to fructose and glucose beverages in children with MASLD (first panel) and without MASLD (second panel). Values are expressed as least-squares means for MASLD (n=12) and non-MASLD (n=14) with standard error bars at each respective time point. *Abbreviations: B, breakfast; L, lunch; D, dinner.*

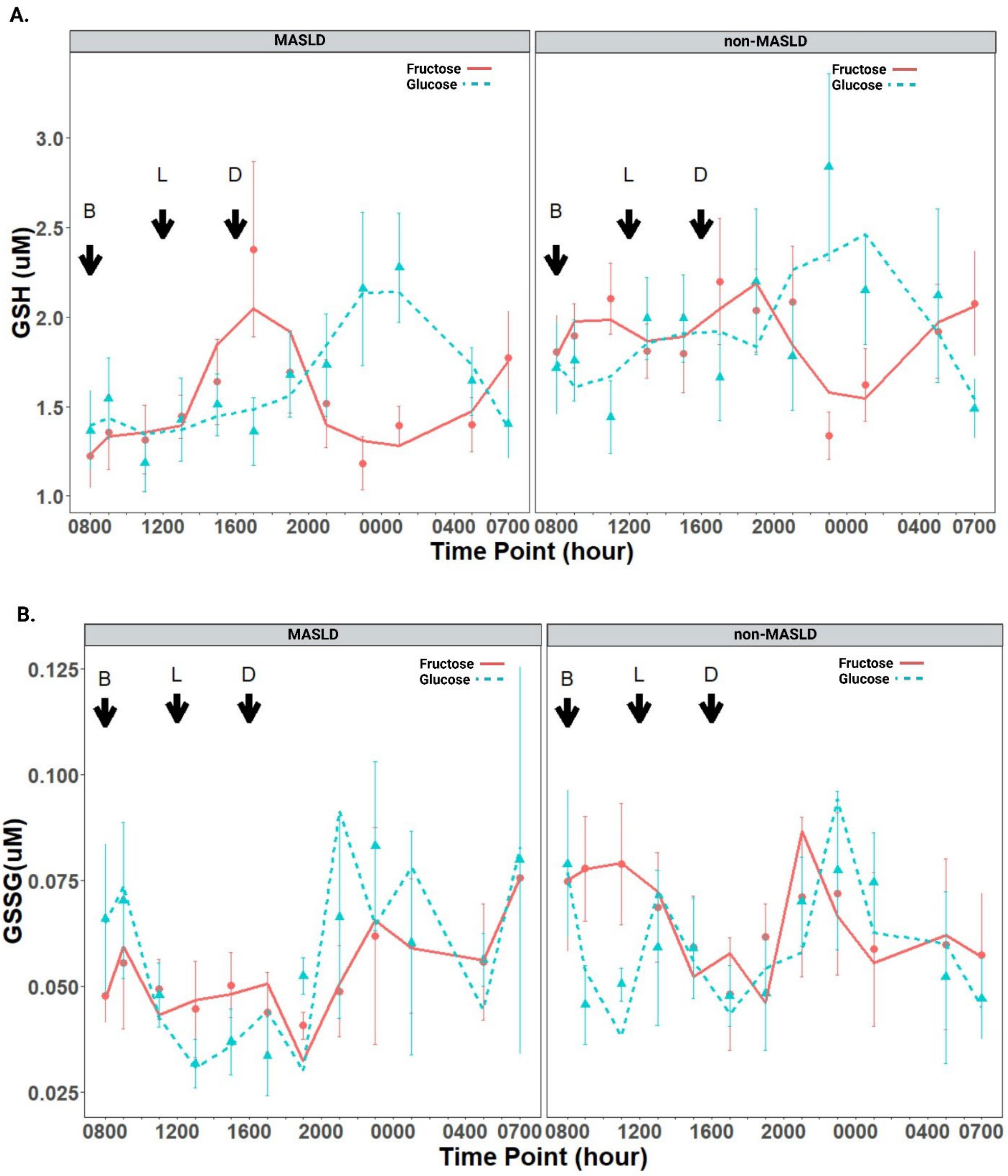

Supplement: Multimedia component 2 [file mmc2.pdf]
